# Real-world experience with doxorubicin and olaratumab in soft tissue sarcomas in England and Northern Ireland

**DOI:** 10.1186/s13569-020-00131-x

**Published:** 2020-05-06

**Authors:** Spyridon Gennatas, Florence Chamberlain, Thomas Carter, Susanna Slater, Elena Cojocaru, Beth Lambourn, Anna Stansfeld, Radha Todd, Mark Verrill, Nasim Ali, Robin L. Jones, Peter Simmonds, Nicola Keay, Heather McCarty, Sandra Strauss, Vassilios Karavasilis, Palma Dileo, Charlotte Benson

**Affiliations:** 1 Sarcoma Unit, Royal Marsden Hospital, Fulham Road, London, SW3 6JJ UK; 2 University College Hospital London, 235 Euston Rd, London, NW1 2BU UK; 3 Hammersmith Hospital, 150 Du Cane Rd, White City, London, W12 0HS UK; 4 Newcastle upon Tyne Hospitals NHS Foundation Trust, Newcastle upon Tyne, UK; 5 The Clatterbridge Cancer Centre NHS Foundation Trust, Birkenhead, UK; 6 Institute of Cancer Research, 15 Cotswold Road, Sutton, London, SM2 5NG UK; 7 University Hospital Southampton, Tremona Rd, Southampton, SO16 6YD UK; 8 Belfast Health and Social Care Trust, Belfast City Hospital, Lisburn Road, Belfast, BT9 7AB UK

**Keywords:** Soft tissue sarcomas, Doxorubicin, Olaratumab, Chemotherapy

## Abstract

**Background:**

A randomised phase II trial demonstrated that the addition of olaratumab to doxorubicin significantly increased overall survival (OS) in patients with advanced soft tissue sarcomas (STS) compared to doxorubicin alone. The recently presented phase III study of doxorubicin and olaratumab in advanced soft tissue sarcoma was discordant with this finding.

**Methods:**

We performed a retrospective analysis of adult patients with advanced-/metastatic STS treated with at least two cycles of doxorubicin and olaratumab at eight sarcoma units across England and Northern Ireland between May 2017 and March 2019.

**Results:**

172 patients were evaluable and 40 patients (23.3%) had died at the time of analysis. Median ECOG performance status (PS) was 1. Median progression free survival (PFS) was 6.8 months (95% CI 5.9–7.7 months). Leiomyosarcoma was the most common histological subtype (75 patients, 43.6%), followed by liposarcomas (19, 11.0%). The mean number of cycles was 5 (doxorubicin range 2–6; olaratumab range 2–23). Two patients (1.2%) had a complete response and 34 (19.8%) had a partial response. 79 (45.9%) had stable and 58 (33.7%) progressive disease. 57 patients (33.1%) experienced grade ≥ 3 neutropenia and 7 patients (4.1%) grade ≥ 3 febrile neutropenia. Grade ≥ 3 anaemia was seen in 21 patients (12.2%). Grade ≥ 3 non-haematological toxicities were seen in 35 patients (20.3%). A clinically significant drop in left ventricular ejection fraction was seen in 6 patients (3.5%). 48 patients (27.9%) required a dose reduction. Overall survival (OS) is pending.

**Conclusions:**

Our results are in keeping with the phase III study findings: response rate, PFS and OS were similar to those reported in the phase III ANNOUNCE trial.

## Background

Doxorubicin with or without ifosfamide is the first line treatment for advanced or metastatic soft tissue sarcomas [1, 2]. Olaratumab is a monoclonal antibody directed against platelet-derived growth factor receptor alpha (PDGFRα), which is responsible for oncogenic signalling, however the precise mechanism of action of olaratumab is likely to be multifactorial [3]. Data from a randomised phase II trial led to accelerated approval by the U.S. Food and Drug Administration (FDA) and conditional marketing authorization by the European Medicines Agency (EMA) of combination doxorubicin and olaratumab in patients with advanced soft tissue sarcomas. The study randomised one hundred and twenty-nine evaluable patients in a 1:1 ratio to either doxorubicin (Day 1) and olaratumab (Day 1 and Day 8) plus doxorubicin or doxorubicin alone (Day 1) for up to eight 21-day cycles. The study met its primary endpoint with improvement in PFS in the combination arm compared to single agent doxorubicin (6.6 months vs 4.1 months) (p = 0.0615; HR 0.67) as well as secondary endpoints of significantly increased OS compared to doxorubicin alone (26.5 months vs 14.7 months (p = 0.0003; HR 0.46)). The most frequently reported adverse event (AE) of any grade was nausea (n = 47, 73%), fatigue (n = 44, 69%), neutropenia (n = 38, 59%) and oral mucositis (n = 34, 53%). Grade ≥ 3 AEs were more frequent with combination treatment compared to doxorubicin alone; fatigue (9.4%), anaemia (12.5%) and neutropaenia (53.2%) were the most frequently reported [4].

The ANNOUNCE phase III study enrolled 509 patients with soft tissue sarcomas with a primary end point of OS. Disappointingly, data from the trial were released in January 2019, and later presented in ASCO in June 2019, which did not support the phase II results. Combination treatment with doxorubicin and olaratumab in patients with advanced soft tissue sarcomas did not meet its primary endpoint in all soft tissue sarcomas including in the leiomyosarcoma sub-group. In this study, starting dose of olaratumab was 20 mg/kg followed by a maintenance dose of 15 mg/kg [5–7].

## Methods

We performed a retrospective analysis of one hundred and ninety patients treated with doxorubicin and olaratumab at eight sarcoma specialist centres in the England and Northern Ireland between May 2017 and March 2019. Local institutional approval was obtained prior to commencing the study. Doxorubicin (75 mg/m^2^) was given on Day 1 of a 21-day cycle and olaratumab (20 mg/kg) on Days 1 and 8 of each cycle. A maximum number of six cycles of doxorubicin were given, as designated by the provisional UK approval for olaratumab. Dexrazoxane was not used in any of these patients. Non-progressing patients continued with maintenance olaratumab until progression or the development of unacceptable toxicity. Inclusion criteria included adult patients with locally advanced/- or metastatic soft tissue sarcomas. All patients had at least 2 cycles (Day 1 with or without Day 8) of olaratumab and 2 cycles (Day 1) of doxorubicin with baseline ECOG performance status (PS) of 0–2. Response was assessed as per RECIST version 1.1 [8]. Kaplan–Meier methods were used to assess PFS as well as descriptive statistics.

## Results

A total of one hundred and ninety patients from eight centres across England and Northern Ireland of which one hundred and seventy-seven were eligible and one hundred and seventy-two were evaluable. Median age at start of treatment was 55.2 years (46.8–63.5 years). There were 96 females (54.2%) and 81 males (45.7%) and median ECOG PS was 1. Leiomyosarcoma was the most common histological subtype (75 patients, 43.6%), followed by liposarcomas (19, 11.0%). A breakdown of all subtypes can be found in Table 1. The median number of metastatic disease sites was 1 (range 0–5) with the most common site of metastasis being the lung (n = 88, 51.2%). The median number of doxorubicin cycles was 5 (range: 2–6) and of olaratumab cycles was 5 (range 2–23).

**Table 1:** Baseline characteristics of 172 eligible and evaluable patients

Characteristic	Total, n = 172
Age at diagnosis (years)	
Median (IQR)	55.2 years (46.8–63.5 years)
Gender	
Female	96 (54.2%)
Male	81 (45.7%)
Soft tissue sarcoma subtype	
Leiomyosarcoma	75 (43.6%)
Liposarcoma	19 (11.0%)
Undifferentiated pleomorphic sarcoma	13 (7.6%)
Synovial sarcoma	10 (5.8%)
Myxofibrosarcoma	8 (4.7%)
Solitary fibrous tissue	6 (3.5%)
Angiosarcoma	5 (2.9%)
Malignant peripheral nerve sheath tumour	5 (2.9%)
Soft tissue sarcoma (NOS)	5 (2.9%)
High grade pleomorphic sarcoma (NOS)	4 (2.3%)
Spindle cell sarcoma (NOS)	3 (1.7%)
Extra skeletal myxoid chondrosarcoma	3 (1.7%)
Endometrial stromal sarcoma	2 (1.2%)
Adenosarcoma	2 (1.2%)
PEComa	1 (0.6%)
Intimal sarcoma	1 (0.6%)
Sites of metastatic disease	
Lung	88 (51.2%)
Liver	31 (18.0%)
Soft tissue	25 (14.5%)
Bone	21 (12.2%)
Pelvis	14 (8.1%)
Abdominal	13 (7.6%)
Peritoneal	11 (6.4%)
Lymph nodes	4 (2.3%)
Cardiac	3 (1.7%)
Intracranial	3 (1.7%)
Renal	2 (1.2%)
Pancreas	1 (0.6%)
Unknown	39 (22.7%)

**Fig. 1:**
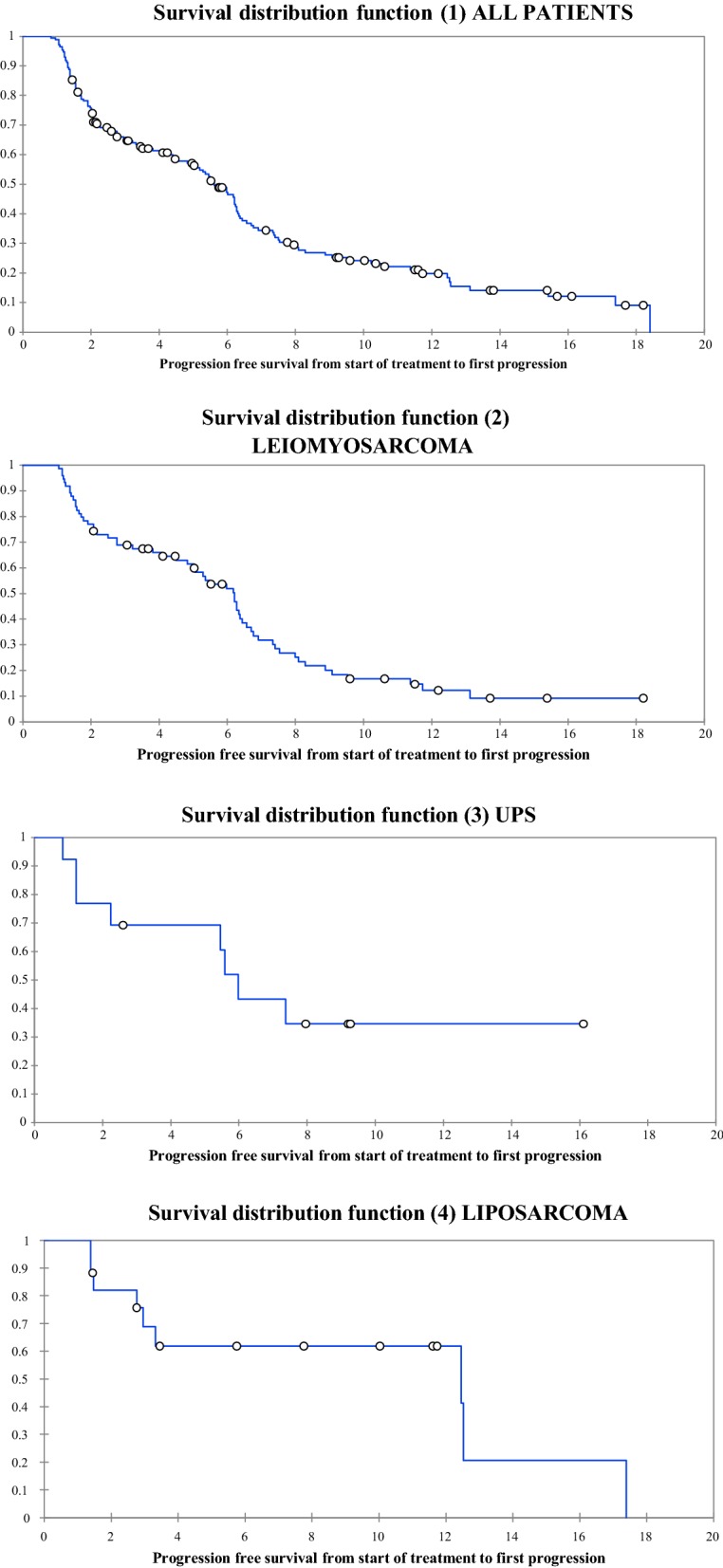
Kaplan-Meier curve for PFS for patients treated with combination doxorubicin and olaratumab (1) all patients (2) leiomyosarcoma (3) undifferentiated pleomorphic sarcoma (UPS) (4) liposarcoma

Median PFS was 6.8 months (95% CI 5.9–7.7 months) for all patients and median PFS for liposarcoma was 9.6 months (95% CI 6.1–13.1). Median PFS for other subgroups is found in Table 2, OS data are not yet mature. One hundred and seventy-two out of 177 had evaluable disease and the overall response rate as per RECIST 1.1 [8] was 36/172 (20.9%). There were two patients (1.2%) with a complete response (CR) [leiomyosarcoma (n = 1), undifferentiated pleomorphic sarcoma (n = 1)]. Thirty-four patients (19.8%) had a partial response (adenosarcoma (n = 2), angiosarcoma (n = 2), leiomyosarcoma (n = 13), myxoid liposarcoma (n = 5), myxofibrosarcoma (n = 1), spindle cell sarcoma (n = 1), synovial sarcoma (n = 4), undifferentiated pleomorphic sarcoma (n = 5)). 79 patients (45.9%) had stable disease. Fifty-eight patients (33.7%) ha progressive disease as their best response. Median follow up from start of treatment to last follow up or death was 245 days (IQR: 131–340 days, SD: 127 days). Forty patients (23.3%) had died at the time of analysis (Fig. 1).

**Table 2:** Progression free survival for patients treated with doxorubicin and olaratumab in our study

Group	Median PFS	95% CI
All patients	6.8	5.9–7.7
Liposarcoma	9.6	6.1–13.1
UPS	5.7	3.8–7.6
Leiomyosarcoma	6.2	5.2–7.2

The two patients with a complete response to doxorubicin and olaratumab were a 51-year-old female with undifferentiated pleomorphic sarcoma and a solitary lung metastasis (Patient A) and a 45-year-old female with leiomyosarcoma with a solitary liver metastasis (Patient B). These patients were treated with six cycles of doxorubicin and eighteen cycles of olaratumab and five cycles of doxorubicin and fourteen cycles of olaratumab respectively. Patient A had experienced a grade 1 anaemia, neutropaenia and thrombocytopaenia during their treatment but no other toxicities or adverse events to treatment. By cycle 18 of olaratumab the lung metastasis had disappeared and treatment was discontinued. This patient was placed on active surveillance and was alive at time of study without evidence of disease. Patient B was treated with doxorubicin and olaratumab after developing metastatic disease in the liver from a retroperitoneal leiomyosarcoma that had been previously resected. Patient B had a partial response to treatment in the liver from treatment but experienced grade four neutropaenia and grade 1 anaemia during their treatment but no other toxicities or adverse events. The response to doxorubicin and olaratumab enabled a partial hepatectomy to be performed following ten cycles of olaratumab. This was a highly necrotic tumour on initial biopsy and remained so on the excision biopsy with no clear evidence of a pathological response in that specimen. Patient B continued olaratumab at time of study with no evidence of disease relapse.

One hundred and sixty-four patients (95.3%) experienced toxicity of any grade. Eighty-two patients (47.7%) experienced a grade ≥ 3 toxicity; 57 patients (33.1%) experienced neutropenia, and 7 patients (4.1%) had febrile neutropenia. Anaemia was seen in 142 patients (82.6%) with grade ≥ 3 anaemia in 21 patients (12.2%). Grade ≥ 3 non-haematological toxicities were seen in 35 patients (20.3%) of whom the most frequently seen toxicity was infection (n = 13, 7.6%), and oral mucositis (n = 6, 3.5%). A clinically significant drop in left ventricular ejection fraction was identified in six patients (3.5%) and one patient (0.6%) required treatment for a myocardial infarction whilst on treatment. Fifty-five patients (32.0%) required hospital admission during their treatment for management of toxicity or complications of treatment. See Table 3 for full details of toxicities.

**Table 3:** Toxicities of patients treated with doxorubicin and olaratumab in our study, phase II and phase III ANNOUNCE study

Grade ≥ 3 adverse events	2019 study (n = 172) (%)	Phase II ANNOUNCE (n = 64) (%)	Phase III ANNOUNCE (n = XX) (%)
*All Grade *≥* 3 AEs*	*82 (47.7%)*	51 (79.7%)	Unknown
Anaemia	21 (12.2%)	8 (12.5%)	Unknown
Neutropaenia	57 (33.1%)	34 (53.2%)	48%
Neutropaenic fever	7 (4.1%)	8 (12.5%)	Unknown
Thrombocytopaenia	3 (1.7%)	Unknown	6%
Hepatotoxicity	2 (1.2%)	Unknown	Unknown
Oral mucositis	6 (3.5%)	2 (3.1%)	3%
Diarrhoea	3 (1.7%)	2 (3.1%)	3%
Nausea	2 (1.2%)	1 (1.6%)	2%
Vomiting	3 (1.7%)	0 (0%)	0%
Fatigue	3 (1.7%)	6 (9.4%)	9%
Cardiac toxicity	6 (3.5%)^a^	1 (1.6%)	Unknown
Sepsis	13 (7.6%)	5 (7.8%)	Unknown

^a^One patient (0.6%) had a myocardial infarction whilst on treatment

Forty-eight patients (27.9%) required a dose-reduction of between 10 and 25% of the recommended starting dose of 75 mg/m^2^ doxorubicin and 20 mg/kg olaratumab either before or during their treatment. The most common reasons for dose reductions were neutropaenia (n = 9, 5.2%), nausea (n = 5, 2.9%), fatigue (n = 5, 2.9%), sepsis (n = 5, 2.9%) and patient co-morbidities (n = 5, 2.9%).

## Discussion

Preliminary results from this real-world, multi-centre retrospective study closely resemble the presented data for the phase III ANNOUNCE [6]. Since these were announced olaratumab has been completely withdrawn from the market as the FDA and EMA have recommended against its use. At the time of study writing one hundred and fifteen patients (66.9%) are alive and OS is pending. There has also been a modest improvement in median OS for patients with advanced and metastatic soft tissue sarcomas from 12 months to 15–19 months over the last decade due to a number of factors [6, 9, 10].

Allowing for the difficulty of making inter-study comparisons, median PFS in our large cohort of patients from across England and Northern Ireland treated with doxorubicin and olaratumab was 6.8 months (compared to 6.8 months in the phase II trial [4]) and fits with the provisional findings from the phase III ANNOUNCE study [5]. Median PFS in the liposarcoma subgroup was slightly higher than the overall cohort 9.6 months but did not meet statistical significance (p = 0.873). However, the liposarcoma subgroup consists of several histopathological subtypes, all with distinct histological features but frequently displaying features of different subtypes within the same mass. Clinical patterns of behaviour can also vary considerably in this subtype [11].

Study limitations included the retrospective nature as well as the range of histological subtypes that were included, reflecting real life clinical experience. However, in the phase III study, dosing of olaratumab differed to the standard dosing used in the United Kingdom and Northern Ireland (20 mg/Kg followed by 15 mg/Kg compared to 20 mg/Kg continuously). We also recognise that overall survival is not yet mature which was the primary endpoint of the phase III ANNOUNCE study [5] and secondary endpoint in the phase II trial [4]. However, this was a large multi-centre study representing the range of patients treated for soft tissue sarcomas across England and Northern Ireland. Despite this we did not identify any subgroup from our cohort that potentially benefited from combination doxorubicin and olaratumab chemotherapy.

Adverse events were similar to that of the phase II trial [4]. The most common grade ≥ 3 AE were neutropaenia (n = 57, 33.1%), and anaemia (n = 21, 12.2%). The frequency of anaemia in our study population was similar to that of the combination arm of the phase II ANNOUNCE study (n = 8. 12.5%). Rates of neutropaenia were higher in the combination arm of the phase II trial compared to our population (n = 34, 53.2%) [4] but this has not been adjusted for the use of granulocyte-colony stimulating factor (G-CSF). Other AEs were reported in similar frequencies in our study. However, we accept that the AE reporting is more stringent within the context of a clinical trial.

In the doxorubicin arm of the GeDDiS trial grade ≥ 3 neutropenia was seen in only 32 of 128 patients (25%) but grade ≥ 3 febrile neutropenia was higher (26 of 128; 20%). Of the non-haematological toxicities, grade ≥ 3 oral mucositis was a lot commoner than in our study (14%). In the GeDDiS trial there was no report of the number of patients that required hospital admission during treatment but only one of 128 (1%) discontinued treatment early due to toxicity. 34 of 128 patients (27%) required a dose reduction, the commonest reasons having been febrile neutropenia and other haematological toxicities [12]. These data do not suggest an increased toxicity profile for the combination treatment.

Although OS data are awaited, the results of our real world multi-centre retrospective study of patients treated with doxorubicin and olaratumab fit with the provisional results of the phase III ANNOUNCE study that PFS is not improved compared to doxorubicin alone [5]. The toxicity profile of the combination treatment was in keeping with published data [4, 5]. Doxorubicin-based therapy remains the first line treatment for most soft tissue sarcomas [1]. Although it is tempting to interpret these findings as showing that liposarcomas may benefit from combination treatment, different liposarcoma subtypes were all grouped together. As these have differing clinical behaviours such a conclusion cannot be drawn safely, and the numbers are too small to look at the individual subtypes.

Over the last decade, three randomized trials have reported single agent doxorubicin as standard first-line therapy for advanced/metastatic soft tissue sarcomas. The GeDDiS trial was a randomised, controlled phase III study that compared gemcitabine and docetaxel with doxorubicin in this setting. Median PFS was 23.3 weeks (95% CI 19.6–30.4) in the doxorubicin group vs 23.7 weeks (95% CI 18.1–20.0) in the gemcitabine and docetaxel group; HR for PFS was 1.28, 95% CI 0.99–1.65, p = 0.06) [12]. This PFS was shorter to the one seen in our study and closer to that in PICASOO III [13]. PICASSO III was a phase III study of doxorubicin and palifosfamide compared to doxorubicin and placebo as first line treatment for patients with advanced soft tissue sarcoma. The primary endpoint of PFS was not met (6.0 vs 5.2 months, hazard ratio 0.86, p = 0.19) as well as the secondary endpoint of OS (15.9 vs 16.9 months, hazard ratio 1.04, p = 0.74) with a higher incidence of grade 3–4 adverse events in the combination arm [13]. Equally in the phase III SARC021 study of doxorubicin and evofosfamide compared to doxorubicin and placebo in the first line as treatment for advanced soft tissue sarcoma, the primary endpoint of OS was not met (18.4 vs 19.0 months, hazard ratio 1.06, p = 0.527) [14]. The above results raise the question as to whether there is any utility in recruiting ‘all comers’ to first line trials in soft tissue sarcoma before exploring if there is a subgroup which might potentially confer benefit and exploring the differences between the populations in the phase II and phase III studies which led to the differing study outcomes.

## Conclusion

Given there has been no improvement in OS and greater toxicity profile compared to single agent doxorubicin, it is difficult to recommend this treatment to patients. At time of writing, the drug manufacturer of olaratumab is suspending promotion of this treatment, and patients may not be initiated on treatment unless participating in a clinical trial or currently using it with clinical benefit [5, 6].

## Data Availability

The datasets used and/or analysed during the current study are available from the corresponding author on reasonable request.

## Abbreviations

AE: Adverse event
ECOG: Eastern cooperative oncology group
EMA: European medicines agency
FDA: Food and drug administration
IQR: Interquartile range
OS: Overall survival
PDGFR: Platelet derived growth factor
PFS: Progression free survival
PS: Performance status
RECIST: Response evaluation criteria in solid tumours
STS: Soft tissue sarcoma
UPS: Undifferentiated pleomorphic sarcoma
